# Control of Corner Separation in Compressor Cascade Using Biomimetic Fish Scales Structure

**DOI:** 10.3390/biomimetics9120746

**Published:** 2024-12-07

**Authors:** Jin-Long Shen, Szu-I Yeh

**Affiliations:** Department of Aeronautics and Astronautics, National Cheng Kung University, Tainan 701, Taiwan; wklaelul@gmail.com

**Keywords:** compressor cascade, fish scale cascade, vortex structure, numerical simulation, total pressure loss

## Abstract

In this study, a fish scale structure with low viscous drag was proposed and applied to the suction surface of a compressor cascade to reduce total pressure loss and suppress corner separation, a key source of compressor inefficiency. By using CFD simulations, the biomimetic structure was identified and integrated into the cascade design. To evaluate its effects, we analyzed secondary flow structures using 2D projected streamlines, axial velocity density (AVD), and vortex visualization techniques. The results show that the fish scale structure effectively reduces the volume of low-energy fluid by 18.36% and decreases total pressure loss at the outlet by 3.5%. Additionally, the AVD iso-surface proved instrumental in identifying low-energy fluid regions, which correlate closely with total pressure loss distribution. These findings highlight the potential of biomimetic-inspired designs to improve compressor performance by mitigating corner separation and reducing flow losses.

## 1. Introduction

The compressor is an essential part of an aircraft engine. Improving its aerodynamic performance and working efficiency is critical for increasing the overall efficiency of an engine [1]. The compressor’s complex internal structure and strong adverse pressure gradient result in significant corner separation on the suction surface of the cascade, negatively impacting engine performance. Wang et al. [2] visualized the vortex structure using the *Q* criterion analysis, revealing that low-momentum fluid in the region of corner separation causes flow losses.

Flow control methods can be applied in the corner region of a compressor. These methods may be active and passive. Active flow control technology includes end-wall suction [3], plasma actuation [4], pulse suction [5], pulse jets [6], and synthetic jets [7]. In this technology, the flow field is controlled by installing auxiliary equipment to introduce energy into the flow field, such as by injecting fluid mass or applying an external force. However, this approach increases the complexity of the blade structure and complicates the manufacture. Passive flow control technology includes vortex generators [8], non-axisymmetric end-wall [9], blade tip winglets [10], and blade ribs [11].

Nowadays, metal additive manufacturing (AM) can be used to produce complex and large-scale titanium alloy component parts with a high degree of flexibility [12,13]. It is widely used in electronics, aerospace, automotive, military, medical, and other fields [14,15,16]. Therefore, metal additive manufacturing technology provides more complex geometry and lightweight design. Various categories of biomimetic technologies are available for reducing total pressure loss in cascade, such as shark skin micro-groove riblet [17], puffer spines [18], Grass Carp scales (i.e., *Ctenopharyngodon idellus* scales) [19,20,21], beetle dimple [22], and Humpback whales flipper with leading-edge tubercles [23]. Therefore, we can use biomimetic microstructures as a novel passive flow control technique to suppress flow separation and reduce flow losses.

According to Meyer et al. [24], in the wind tunnel, using a single vortex generator significantly reduces the corner separation between the suction surface and the guide vane. It improves the aerodynamic performance of the compressor. Hergt et al. [25] utilized both experimental and numerical approaches with three different vortex generator types of vortex generators positioned on the end-wall and blade to improve the corner separation and reduce the flow loss. Diaa et al. [26] studied the effect of double and wishbone vortex generators on the aerodynamic performance of compressor cascades. These vortex generators eliminated or delayed separation on the blade suction surface and end-wall but slightly increased the total pressure loss. Hu et al. [27] developed a combined flow control strategy that integrates a blade slot and vortex generator within a single-stage axial transonic compressor, aiming to enhance the compressor’s total pressure ratio and efficiency, thereby improving its performance and stability. Benner et al. [28,29] reviewed the literature on the modeling of secondary loss in turbines. They proved a new empirical method for predicting the penetration depth of the corner separation at the blade trailing edge along the spanwise direction. The correlation shows a strong agreement between the predicted values and the experimental results. Wang et al. [30] employed an advanced vortex generator technique to design an L-shaped groove on the end-wall of the compressor cascade. The vortex generated by the L-shaped groove acted as a barrier to the end-wall cross flow, reducing the interaction of the end-wall fluid with the suction side of the blade. This design ultimately minimized the spanwise penetration depth.

The main objective of this study is to design a low-drag microstructure laid in front of the corner vortex to produce a function like a vortex generator [8,31] to control or suppress the corner separation. Therefore, we chose the grass carp scale structure, inspired by the studies of microstructure, such as shark skin micro-riblets [32]. This design is more straightforward than the shark structure, requiring only one row of scales to improve the flow field. This study provides a design concept that uses a flat plate with a biomimetic microstructure to identify geometric parameters that achieve a low viscous drag reduction rate. The microstructure will be applied to the blade’s suction surface to decrease the cascade’s total pressure loss coefficient. This design idea will help develop more types of microstructures to suppress the corner separation of the cascade and avoid a reduction in compressor efficiency.

## 2. Numerical Investigation

### 2.1. The Design Concept

**Figure 1:**
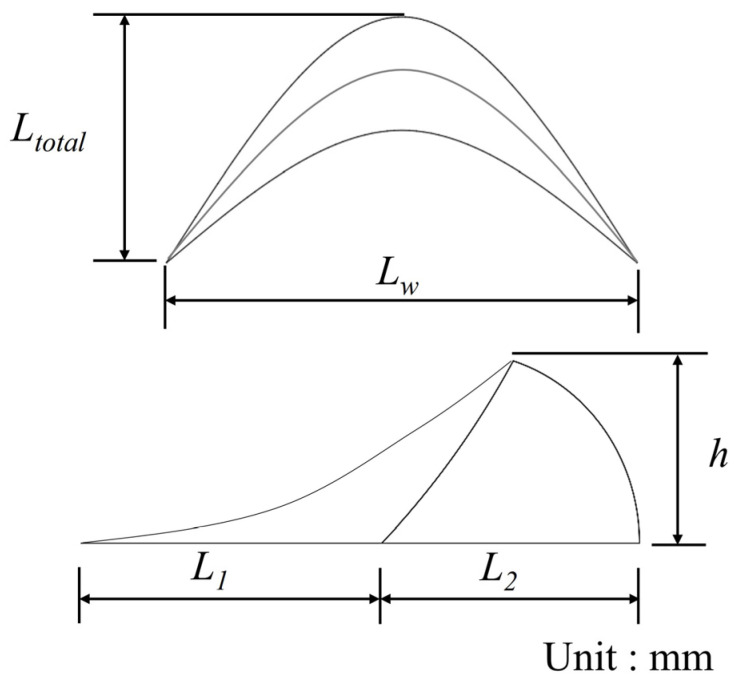
Fish scale geometry.

Drag reduction can be achieved through biomimetic structures, which reduce drag by altering the geometric shape of an object without requiring additional equipment or energy consumption. Wu et al. [21,22] showed that the fish scale surfaces were highly effective for drag reduction at low flow rates. The drag reduction rate clearly decreased as the flow rate increased. Compared with a flat plate, the fish scale-inspired micro-crescent patterned model had a maximum drag reduction of 3.047% (at 0.66 m/s). In this study, we further investigate the drag reduction effects of grass carp scale structures with different geometric configurations and analyze the underlying mechanisms from a flow field perspective. A schematic of the scale structures used is shown in Figure 1. We selected different fish scale heights h = 0.4, 0.5, 0.6, 0.7, 0.8 mm, and spacings $L_{s}$ = 2, 3, and 4 mm to analyze which geometric parameters have a low viscous drag reduction rate, which is all shown in Table 1.

### 2.2. The Computational Domains

In this study, we utilized two models: a flat plate model and a cascade model. The flat plate model was specifically designed to investigate the flow field effects induced by the fish scale structure and served as the geometric shape of the passive component to control corner separation in the cascade model. The cascade model was further employed to analyze the secondary flow losses and cascade performance by the fish scale structure when applied to compressor blades. A detailed description of the computational domains for both models is provided below.

#### 2.2.1. Computational Domain for the Flat Plate

**Figure 2:**
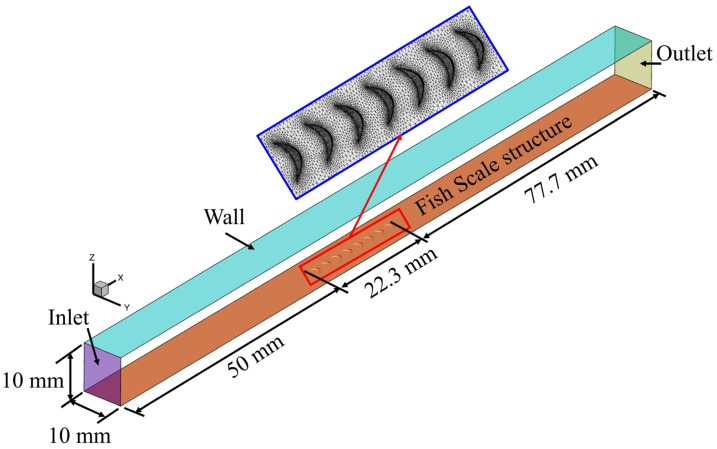
The computational domain of the plate with fish scale structure.

In this study, one fish scale array was placed on the surface plate, and a three-dimensional (3D) computational domain along the flow direction was used to simulate and analyze the fluid flow situation in the near-wall region (Figure 2). The calculation domain is intended to compare the fish scale surface with a smooth surface, under the assumption that the upper surface is smooth while the lower surface resembles fish scales. Moreover, the smooth region of a relative length is added before the inlet and outlet. The computational domain is length (x) of 150 mm, width (y) of 10 mm, and height (z) of 8.5 mm. The numerical simulation for a flat plate set up, the Shear Stress Transport *k*-*ω* (SST *k*-*ω*) turbulent model, is selected. The inlet uses the velocity inlet boundary (the same as the experimental measurement velocity), and the outlet is the atmosphere boundary. Meanwhile, the left and right walls are both symmetric boundaries.

#### 2.2.2. Computational Domain for Compressor Cascade

**Figure 3:**
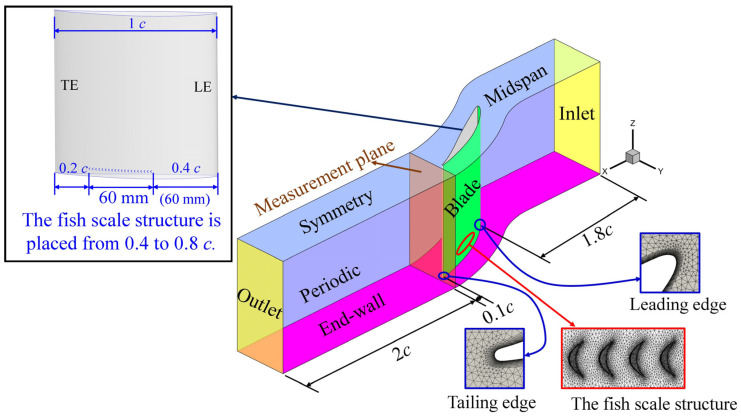
Computational domain of compressor cascade with fish scale structure.

The cascade blades are of NACA 65810 [33,34]; Table 2 shows the specifications for the blade types of the parameters. The calculation uses a computational domain for half of the experimental flow domain in the spanwise direction, with symmetry boundary conditions applied in the mid-span, as shown in Figure 3. Experimentally measured velocity and turbulence intensity are prescribed at the inlet. The outlet boundary condition is set as uniform atmospheric pressure. The blade and end-wall were configured as adiabatic no-slip boundary conditions, and the interface on the two sides of the domain is characterized as translational periodic boundaries. To suppress corner separation, some studies [24,25,26,27] indicated designing a vortex generator in front of the corner. Therefore, we want to create a fish scale structure array to replace the vortex generator. The fish scale structure is placed from 0.4 *c* to 0.8 *c* on the suction surface.

### 2.3. Grid Independence Analysis

ANSYS Fluent [35] has been used in this study to conduct numerical simulation analysis of a flat plate and the cascade. The fish scale structure and near-wall region grid have been refined to precisely capture the complex flow structure in the near-wall region (i.e., for the SST *k*-*ω* turbulence model, ensure that y^+^ close 1).

**Figure 4:**
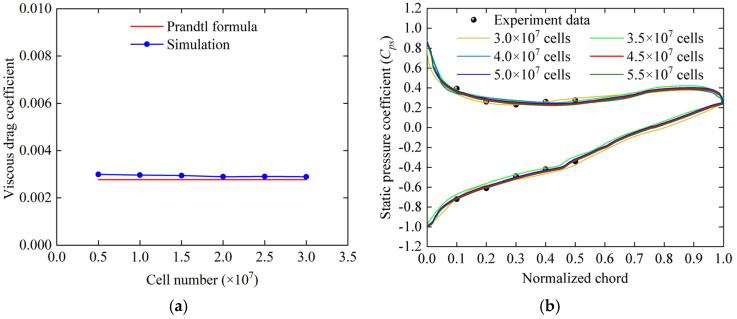
Grid independence analysis. (**a**) Comparison of the viscous drag coefficient of the smooth plate with the Prandtl theory formula; (**b**) comparison of the static pressure coefficient distribution between numerical and experimental results.

The influence of different grid numbers (i.e., cell numbers) on the viscous drag coefficient of the smooth plate and the static pressure coefficient (*C_ps_*) on the blade surface was investigated. The results show that for a smooth plate with a grid of more than 2 million cells, the error in the viscous drag coefficient compared to the Prandtl theory value is relatively small (Figure 4a). For the cascade simulations, the distribution of the static pressure coefficient on the blade surface for grids is over 4.5 million, as shown in Figure 4b.

### 2.4. Numerical Method and Analysis

#### 2.4.1. Viscous Drag Coefficient for the Plate

In the plate simulation, the viscosity drag coefficient (*C_f_*), which was defined as,

(1) $$C_{f}=\frac{2\times \tau_{w}}{\rho \times V^{2}}$$

where $\tau_{w}$ is the calculated wall shear stress (N/m^2^), ρ is the density (kg/m^3^), V is the inlet velocity (m/s), and A is the projected area (m^2^).

For comparison, the viscous drag coefficient (*C_F_*) was also calculated using the Prandtl [18,36] turbulent boundary layer formula as follows:

(2) CF=0.074Re0.2

The *C_f_* and *C_F_* are listed in Table 3; the errors of these two parameters are all less than 4.54%, suggesting a sufficient level of accuracy for engineering applications.

The viscous drag reduction rate (*DV*) is used to evaluate the fish scale structure performance. The equation is as follows:

(3) $$DV=\frac{F_{smooth}-F_{viscous}}{F_{smooth}}\times 100\%$$

where $F_{smooth}$ is the viscous drag for the smooth bottom surface (N), and $F_{viscous}$ is the viscous drag to the fish scale surface (N).

#### 2.4.2. Static Pressure Coefficient for Cascade

**Figure 5:**
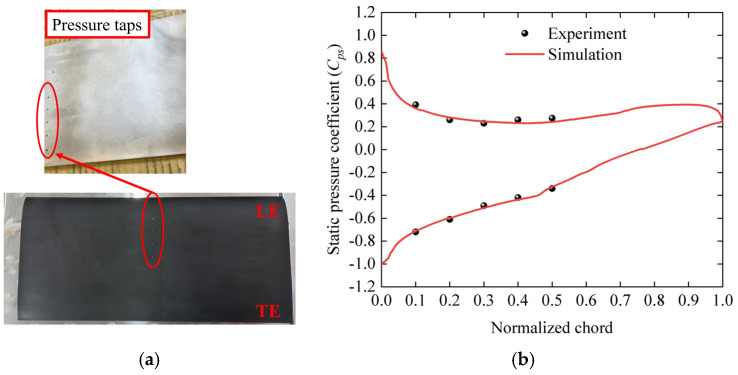
Static pressure coefficient experiment. (**a**) Pressure taps on the suction surface of the blade; (**b**) comparison of the static pressure coefficients from simulation and experiment.

The low-speed cascade wind tunnel at National Cheng Kung University is being used as the wind tunnel experiment for the present work with a wind speed of 20 m/s. The static pressure is measured through the pressure tap (as shown in Figure 5a) in the range of 10% to 50% of the chord length of the middle blade, and then the static pressure is substituted into the equation to calculate the static pressure coefficient (*C_ps_*).

(4) $$C_{ps}=\frac{P_{s,local}-P_{s,in}}{P_{t,in}-P_{s,in}}$$

where $P_{t,in}$ and $P_{s,in}$ represent the total pressure and static pressure at inlet flow, respectively. $P_{s,local}$ represents the static pressure at the pressure tap.

Figure 5b presents the experimental and simulated distributions of the static pressure coefficient on the blade surface. It can be found that the static pressure coefficient on the suction surface of the blade is similar to our experimental data. The results verify the high accuracy of the numerical model.

#### 2.4.3. Total Pressure Coefficient

The total pressure loss coefficient (*C_pt_*) indicates the total flow loss of the cascade and is defined as follows:

(5) $$C_{pt}=\frac{P_{t,in}-P_{t,local}}{P_{t,in}-P_{s,in}}$$

where $P_{t,in}$ and $P_{s,in}$ are the total pressure and static pressure at inlet flow, and $P_{t,local}$ represents the local total pressure.

#### 2.4.4. Vortex Visualization

The biomimetic structure significantly impacts the flow structures, accompanied by changes in the flow loss and the cascade performance.

The axial velocity density (AVD) indicates the size of the low-energy fluid region within the volume enclosed by an iso-surface and is calculated as follows:

*AVD* = *ρ* × *V_a_* (6)

where *ρ* and *V_a_* are the local density and axial velocity, respectively.

The three-dimensional vortex structure in the passage can be visualized by iso-surfaces of the *Q* criterion [37].

The *Q* criterion can be defined as follows:

(7) $$Q=\frac{1}{2}\left({\left\|\Omega \right\|}^{2}-{\left\|S\right\|}^{2}\right)$$

(8) $$\frac{\partial u_{i}}{\partial x_{j}}=S+\Omega$$

(9) $$S=\frac{1}{2}\left(\frac{\partial u_{i}}{\partial x_{j}}+\frac{\partial u_{j}}{\partial x_{i}}\right)$$

(10) $$\Omega =\frac{1}{2}\left(\frac{\partial u_{i}}{\partial x_{j}}-\frac{\partial u_{j}}{\partial x_{i}}\right)$$

where *S* is the strain rate tensor, Ω is the vorticity tensor, and *Q* is the velocity gradient tensor. When a *Q* value greater than 0 indicates the existence of a vortex, by definition, the *Q* criterion defines vortices as regions where the magnitude of rotation is greater than the magnitude of the strain rate.

## 3. Results and Discussion

### 3.1. Drag Reduction Evaluation of Plate

**Figure 6:**
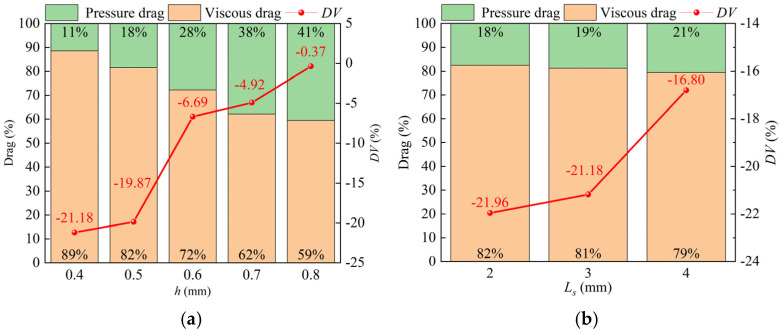
The viscous drag reduction rate (*DV*). (**a**) The *DV* for different fish scale heights; (**b**) the *DV* with different spacing (*L_s_*) when the scale height *h* = 0.4 mm.

The viscous drag reduction rate (*DV*) is calculated to compare the viscous drag of different biomimetic structures with that of a smooth surface, as shown in Figure 6a. The green area represents the pressure drag, while the orange area represents the viscous drag, showing their respective proportions for each case. The red line represents the reduction rate of the viscous drag (*DV*) compared to the original case. Therefore, the *DV* truly reflects the reduction rate of the viscous friction drag caused by the fish scale structure. The viscous drag reduction rate decreases with a reduced fish scale structure height (*h*), with the *DV* value showing the most significant decrease of 21.18% when the fish scale height is *h* = 0.4 mm. Therefore, the authors selected a fish scale height of *h* = 0.4 mm and varied the spacing (*L_s_*), then tried to find the *L_s_* for the lowest *DV* value, as shown in Figure 6b. We can observe from the figure that the *DV* lowest is 21.96% when *L_s_* = 2 mm compared to a smooth plate. The analysis results indicate that the fish-scale structure demonstrates a significant effect in reducing the viscous drag.

**Figure 7:**
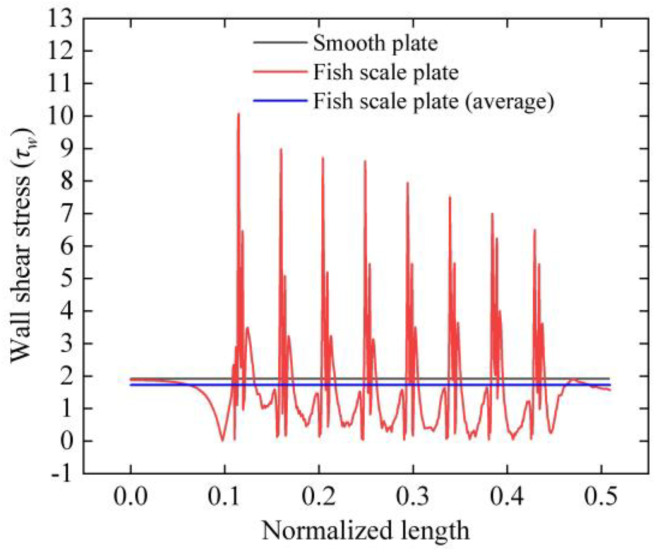
Wall shear stress on a smooth plate compared to a fish scale plate.

The black and red lines in Figure 7 show the wall shear stress distribution of the X–Z cross section with Y = 5 mm for the smooth plate and the fish scale plate, respectively. The blue line is the average wall shear stress of the fish scale plate. As the fluid flows through the first fish scale structure, the wall shear stress rises rapidly, reaching a maximum at the top of the structure. Then, the fluid element flows through the grooves between the fish scales to achieve the lowest wall shear stress. Although the fish scale structure dramatically increases the peak value of the wall shear stress at the top of the element, the overall average wall shear stress is much lower than that of a smooth plate. Overall, the wall shear stress on the fish scale plate (1.725 N/m^2^) is lower than that on the smooth plate (1.915 N/m^2^).

**Figure 8:**
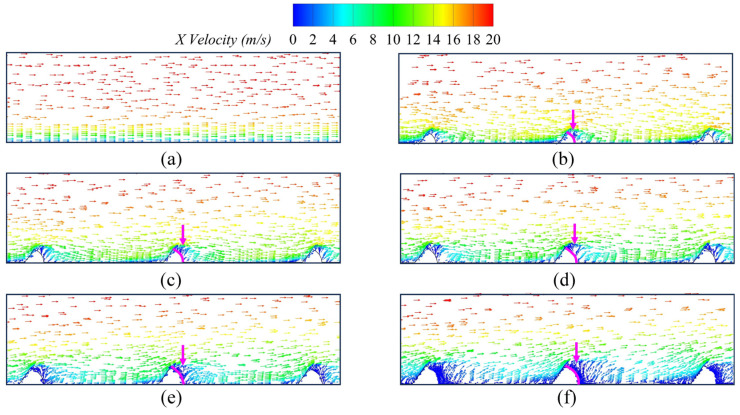
Velocity vectors diagram of the smooth plate and fish scale plate. (**a**) Smooth plate; (**b**) *h* = 0.4 mm; (**c**) *h* = 0.5 mm; (**d**) *h* = 0.6 mm; (**e**) *h* = 0.7 mm; (**f**) *h* = 0.8 mm.

**Figure 9:**
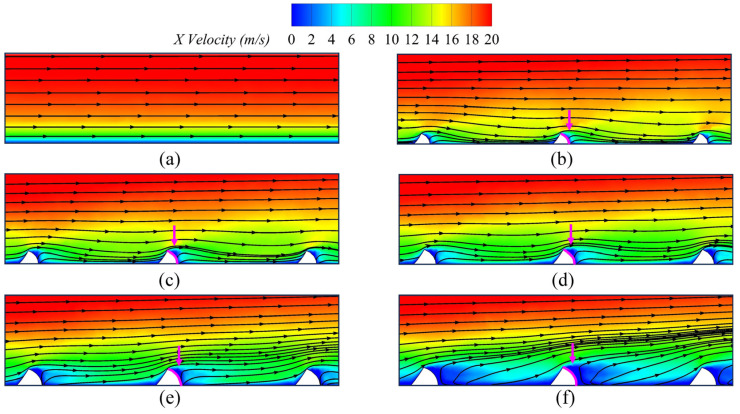
Streamlines and velocity contours diagram of the smooth plate and fish scale plate. (**a**) Smooth plate; (**b**) *h* = 0.4 mm; (**c**) *h* = 0.5 mm; (**d**) *h* = 0.6 mm; (**e**) *h* = 0.7 mm; (**f**) *h* = 0.8 mm.

Figure 8 shows the velocity vector diagram of the X–Z cross-section in Y = 3 mm, where the pink arrow indicates the back surface of the fish scale structure on the wall surface. We can observe that the climbing vortex forms on the back surface of the fish scale structure, thereby reducing the viscous drag of the wall surface in these regions. To further investigate the influence of the fish scale structure on low-energy fluid regions, the authors drew the velocity diagram and the projected 2D streamlines in the fish scale structure region at Y = 3 mm in the X–Z cross-section, as shown in Figure 9. An increase in fluid velocity is observed in the grooves between two fish scales when the scale height is 0.4 mm, which contributes to reducing the low-energy fluid region. This observation underpins the choice of a fish scale height of 0.4 mm for application on the suction surface of the cascade. Combining Figure 7 and Figure 8, the fluid formed a climbing vortex on the downstream surface between adjacent fish scale structure elements. The climbing vortex raised the kinetic energy of part of the fluid, reducing the wall shear stress.

### 3.2. Flow Field Analysis of Cascade

**Figure 10:**
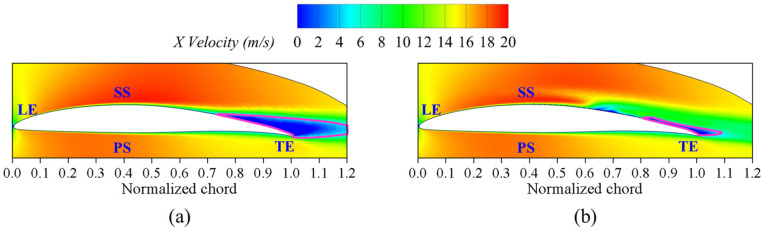
X-Velocity distribution at Z/s = 4% blade height. (**a**) ORI; (**b**) fish scale cascade.

**Figure 11:**
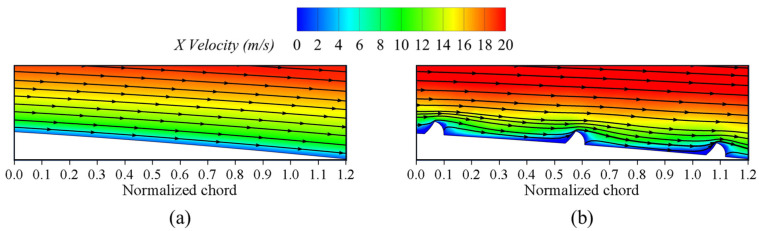
Local flow structure at Z/s = 4% blade height. (**a**) ORI; (**b**) fish scale cascade.

To investigate the flow loss associated with the fish scale structure placed between 40% and 80% of the cascade chord length, we examined the velocity distribution and projected 2D streamlines. Figure 10 represents the blade height Z/s = 4% cross-sectional velocity distribution, and Figure 11 depicts the local flow field structure and projected 2D streamlines of the fish scale cascade. By combining the analyses of Figure 10 and Figure 11, it is evident that the climbing vortex induced by the fish scale structure energizes the nearby flow field. This process transfers energy from the wall boundary layer into the passage vortex (PV) region, increasing the kinetic energy of the low-energy fluid within the PV. As a result, the intensity of the PV region is reduced.

**Figure 12:**
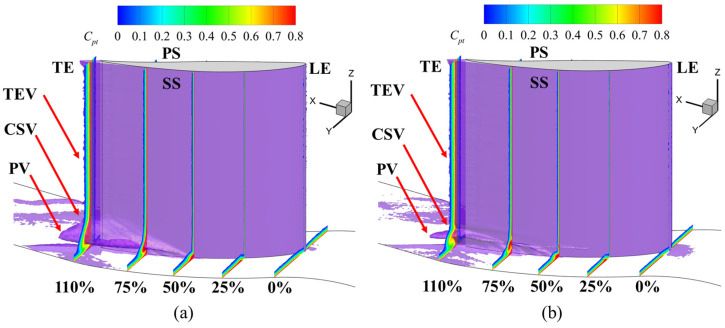
The total pressure loss coefficient in subsequent sections is normal to the streamwise direction. (**a**) ORI; (**b**) fish scale cascade.

In the flow field, the fluid near the leading edge of the suction surface or the end-wall experiences viscous effects that reduce its velocity. Additionally, as the fluid passes through vortex structures, secondary flow losses further decrease its kinetic energy. Therefore, the axial velocity density (AVD) iso-surface effectively reveals low-energy fluid regions influenced by boundary layer effects and secondary flow losses [38]. Understanding the suppression of the accumulation of low-energy fluid in the corner region and reducing the range of the high-loss region is investigated. Figure 12 shows the evolution of the total pressure loss coefficient along the X-direction in the channel, highlighting the low-energy fluid region at the corner region, which is characterized by the iso-surface with an AVD value of 5 kg/(s·m^2^). The low-energy fluid region in the channel is mainly located at the trailing edge of the blade in the near-end-wall regions; some are also at the corner region near the suction surface of the blade. Therefore, the fish scale structure placed in front of the corner vortex can increase the kinetic energy of the fluid around the corner region, producing an effect similar to that of a vortex generator. The volume of low-energy fluid in the cascade describes the flow losses in the passage channel. The fish scale structure reduces this volume by 18.36%. Comparing the fish scale cascade case with the ORI case, it can be found that the low-energy fluid is significantly reduced at the positions of the passage vortex (PV) and the concentrated shedding vortex (CSV), which can improve the flow separation in the corner region. In addition, the fish scale structure reduces the accumulation range of low-energy fluid in the suction corner region, thereby reducing the total pressure loss.

**Figure 13:**
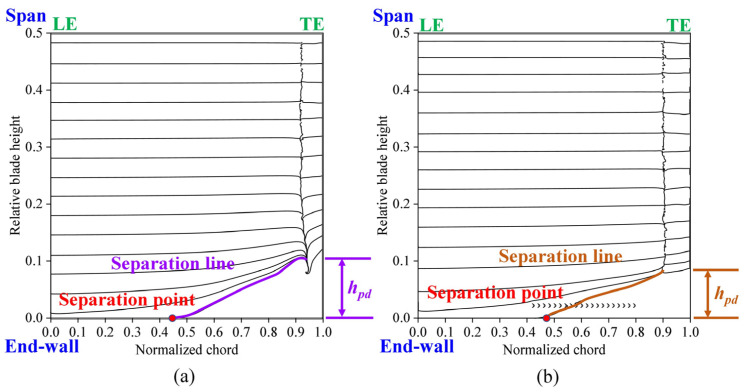
Numerical limiting streamlines on the suction surface of the cascade. (**a**) ORI; (**b**) fish scale cascade.

Figure 13 compares the limiting streamlines on the suction surface for the ORI and fish scale cascade cases. The interaction between the cross flow in the passage channel and the suction surface of the blade suppresses the climb secondary flow, reducing the spanwise penetration depth (marked by *h_pd_*). Combining Figure 12 and Figure 13, the passage vortex structure is weakened due to less end-wall fluid being transported by the climb secondary flow in the fish scale structure. These cases have lower energy fluid regions, lower total pressure loss, and lower spanwise penetration depth. This result is consistent with the phenomena described by Benner et al. [28,29] and Wang et al. [30].

**Figure 14:**
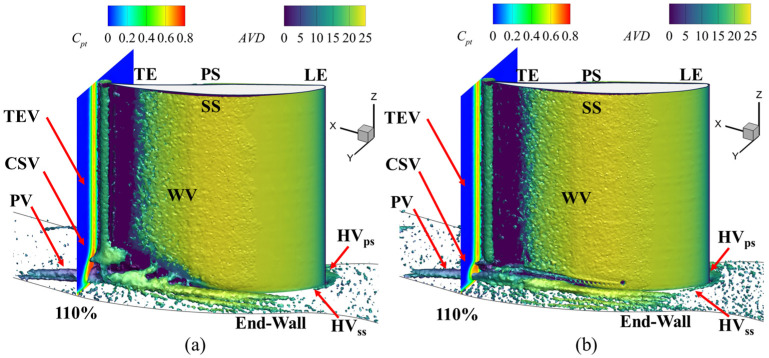
The 3D vortex structure in the corner region and total pressure loss at the outlet of the cascade. (**a**) ORI; (**b**) fish scale cascade.

Many vortex structures were observed in the flow field, including the corner vortex (CV), passage vortex (PV), concentrated shedding vortex (CSV), wall vortex (WV), and trailing-edge vortex (TEV). Therefore, we examined the mechanisms underlying the effect of the fish scale structure on the aerodynamic performance of the cascade. Figure 14 illustrates the three-dimensional (3D) vortex structure around the cascade corner region. This structure is represented by *Q* = 4 × 10^4^ s^−2^ and is colored by the AVD parameter.

Because of the formation of an adverse pressure gradient, the pressure surface (PS) branch with the horseshoe vortex (HV) gradually developed from the leading edge (LE) of blades adjacent to the PV. The WV caused by the PV appeared on the suction surface (SS). In addition, the CSV appeared at the trailing edge (TE). The figure shows that the fish scale structure effectively reduces the size of the corner vortex in both the spanwise and pitchwise directions and suppresses its strength. Combining Figure 13 and Figure 14, it is evident that the separation point shifts downstream, and the separation line extends less toward the end-wall, indicating a reduced spanwise penetration depth. These changes result in lower flow losses and total pressure loss in the fish scale cascade case compared to the ORI case [30]. Moreover, it decreases the height of the CSV at the trailing edge. It reduces the high-loss region along the spanwise direction, thereby decreasing the total pressure loss at the outlet of the blade. Summarizing the above results, we can see that if the fish scale structure is placed within 40% to 80% of the chord, it weakens the structure of the PV, and CV can be reduced.

The performance of the cascade is affected by flow loss due to the viscous effect of the fluid. Significant losses occur in the vortex, where a concentrated velocity gradient (vorticity) is present. Flow losses are strongly dependent on the development of the boundary layer and the vortex structure within the cascade.

**Figure 15:**
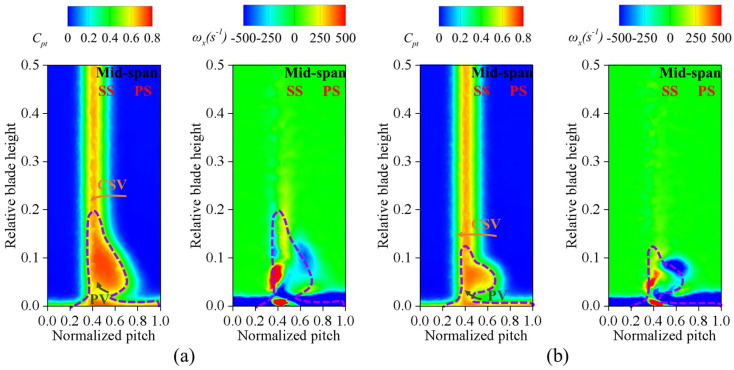
Total pressure loss coefficient, X-vorticity, and secondary flow structure distribution at the cascade outlet. (**a**) ORI; (**b**) fish scale cascade.

Figure 15 shows the distributions of total pressure loss and X-vorticity at the outlet of the cascade. By comparing the X-vorticity and total pressure loss contours, we can see that the fish scale structure decreases the PV structure and CSV. In particular, the PV and CSV are affected by the fish scale structure, resulting in a reduction in these vortex structures and a movement toward the end-wall. As a result, the fish-scale-structured cascade case has a 3.5% reduction in total pressure loss at the outlet compared to the ORI case.

## 4. Conclusions

This study addresses the critical challenge of reducing secondary flow losses in compressors through the innovative application of biomimetic structures. By incorporating a fish scale-inspired design on the suction surface of the cascade model, this research explores how biologically inspired features can enhance aerodynamic performance and efficiency. Numerical simulations reveal that the fish scale structure, strategically placed upstream of the corner vortex, induces a climbing vortex between scales, effectively suppressing corner separation and minimizing pressure losses.

The results show that the fish scale structure reduces viscous friction drag on the wall by 21.96% compared to a smooth plate, while also affecting passage vortex and concentrated shedding vortex behavior. These changes result in a 3.5% reduction in total pressure loss at the outlet compared to the original cascade. Additionally, cases with shorter spanwise penetration depths exhibit smaller low-energy fluid regions and further reductions in total pressure loss. The use of AVD effectively visualizes these low-energy regions, providing an efficient method to identify areas of higher total pressure loss. These findings underline the potential of biomimetic structures to improve compressor cascade performance, offering a promising approach for enhancing efficiency and stability in turbomachinery.

## Figures and Tables

**Table 1:** Fish scale parameter.

Case	Scale Height (h) [mm]	Scale Width ($L_{w}$) [mm]	Scale Length ($L_{total}$) [mm]	Scale Front Length ($L_{1}$) [mm]	Scale Back Length ($L_{2}$) [mm]
1	0.4	2.5	1.3	0.7	0.6
2	0.5	3.125	1.625	0.875	0.75
3	0.6	3.75	1.95	1.05	0.9
4	0.7	4.375	2.275	1.225	1.05
5	0.8	5	2.6	1.4	1.2

**Table 2:** Cascade parameters.

Parameter	Value
Blade span, s [mm]	315
Chord length, c [mm]	150
Pitch, t [mm]	78
Solidity, σ=c/t	1.92
Inlet flow angle, $\beta_{1}$ [°]	45
Outlet flow angle, $\beta_{2}$ [°]	22.5
Angle of attack, α [°]	15
Inlet velocity [m/s]	20

**Table 3:** Verification results.

Viscous Drag Coefficient (*C_F_*)	Viscous Drag Coefficient (*C_f_*)	Error (*C_f_* − *C_F_*)/*C_F_* × 100%
0.002766	0.002891	4.54%

## Data Availability

The data supporting this study’s findings are available from the corresponding author upon reasonable request.
